# A Comprehensive Review of the Clinical Evidence on the Efficacy, Effectiveness, and Safety of Silodosin for the Treatment of Benign Prostatic Hyperplasia

**DOI:** 10.7759/cureus.85445

**Published:** 2025-06-05

**Authors:** Omar S Akhtar, Vishal Singh, Komel A Bhojani, Shruti Dharmadhikari, Chaitany Bhargave, Amey Mane, Suyog Mehta

**Affiliations:** 1 Urology, Hakim Sanaullah Specialist Hospital, Sopore, IND; 2 Medical Affairs and Clinical Research, Sun Pharmaceutical Industries Ltd., Mumbai, IND; 3 Medical Affairs and Clinical Research, Sun Pharma Laboratories Limited, Mumbai, IND

**Keywords:** alpha-1 adrenoceptor blockers, benign prostatic hyperplasia, cardiovascular, comorbidities, effectiveness, efficacy, lower urinary tract symptoms, safety, silodosin

## Abstract

Silodosin, a highly selective α1A-adrenoceptor blocker, is an effective treatment for lower urinary tract symptoms (LUTS) associated with benign prostatic hyperplasia (BPH). Despite its growing use, there remains a lack of comprehensive evidence evaluating the efficacy/effectiveness and safety of silodosin in BPH patients, especially in patients with comorbidities. This review aimed to synthesize recent clinical evidence on the efficacy/effectiveness and overall safety of silodosin in treating BPH. The PubMed literature search identified 23 articles (clinical trials: n = 18; observational studies: n = 5) published between 2014-2024 for inclusion. Silodosin alone (n = 4) significantly improved urological outcomes and alleviated LUTS in BPH patients. In total, 19 studies examined the efficacy/effectiveness of silodosin compared with placebo (n = 2) or various pharmacological treatments (n = 17) for BPH, including other α1-adrenoceptor blockers and combination therapies. Silodosin reduced nocturia in BPH patients (n = 5). In BPH patients with comorbidities such as heart diseases, silodosin alleviated LUTS and enhanced quality of life (n = 5). Across 21 studies evaluating safety, silodosin was well-tolerated; the frequency of retrograde ejaculation/ejaculatory disorder ranged from 0.85% to 34.4%. Notably, the selective action of silodosin on α1A-adrenergic receptors was associated with minimal cardiovascular adverse events. This review demonstrated that silodosin is efficacious/effective and well-tolerated in BPH patients, including patients with cardiovascular comorbidities.

## Introduction and background

Benign prostatic hyperplasia (BPH) is among the most common urologic conditions in males, and its prevalence increases with age [1,2]. In 2000, the global prevalence of BPH was 51.1 million (95% uncertainty interval = 43.1-69.3), which increased to 94.0 million (95% UI = 73.2-118) in 2019 [2]. In India, the prevalence was reported to be 18.2 million in 2019 [2]. BPH is characterized by a non-cancerous enlarged prostate and is associated with lower urinary tract symptoms (LUTS), such as frequent urination, nocturia, and incomplete voiding [3]. These symptoms significantly impact patients’ quality of life (QoL), necessitating prompt medical attention [4].

Globally, α1-adrenoceptor blockers (α1-blockers) are considered the first-line treatment for patients with bothersome, moderate-to-severe LUTS associated with BPH by the European Association of Urology (EAU) [5], the American Urological Association (AUA) [6], and the Urological Society of India (USI) [7]. In India, commonly prescribed α1-blockers for the clinical management of BPH include tamsulosin, alfuzosin, and silodosin [8]. Molecular studies show that α1A-adrenoceptors constitute about 69.3% of α1 receptors in normal prostates and up to 85% in BPH-affected prostates. Silodosin exhibits an exceedingly high α1A:α1B binding ratio of 162:1, indicating strong selectivity for α1A receptors predominantly located in the prostate and lower urinary tract. This high uroselectivity allows silodosin to reduce smooth muscle contraction in these areas, improving urinary symptoms while minimizing effects on blood pressure. Because it spares α1B receptors mainly found in vascular tissue, silodosin has a favorable cardiovascular safety profile. Thus, silodosin effectively relieves LUTS with minimal cardiovascular side effects [9].

The aging population of India is increasing [10], and with it, the burden of BPH is expected to increase, especially in patients with comorbidities [11]. Halder Sr. et al. (2023) reported that the chances of having BPH were directly proportional to the number of comorbidities [11]. Compared to individuals without comorbidities, those aged >60 years with ≥4 comorbidities were nearly six times more likely to have BPH. Understanding the current disease trends and treatment landscape is critical to managing BPH in older adults and addressing the growing burden in India. Despite the increasing use of silodosin, there remains limited evidence on its efficacy and safety in BPH patients with common comorbidities such as hypertension and diabetes, as well as in those refractory to other α-blockers. Additionally, data on silodosin’s cardiovascular safety profile and its effectiveness in real-world clinical practice over the last decade are sparse. This narrative review aims to fill these gaps by consolidating and critically evaluating clinical studies from the past 10 years on silodosin’s role in treating BPH, particularly focusing on its use in patients with comorbidities and those who have shown inadequate response to other treatments.

## Review

Methodology

Literature Search Strategy

**Figure 1:**
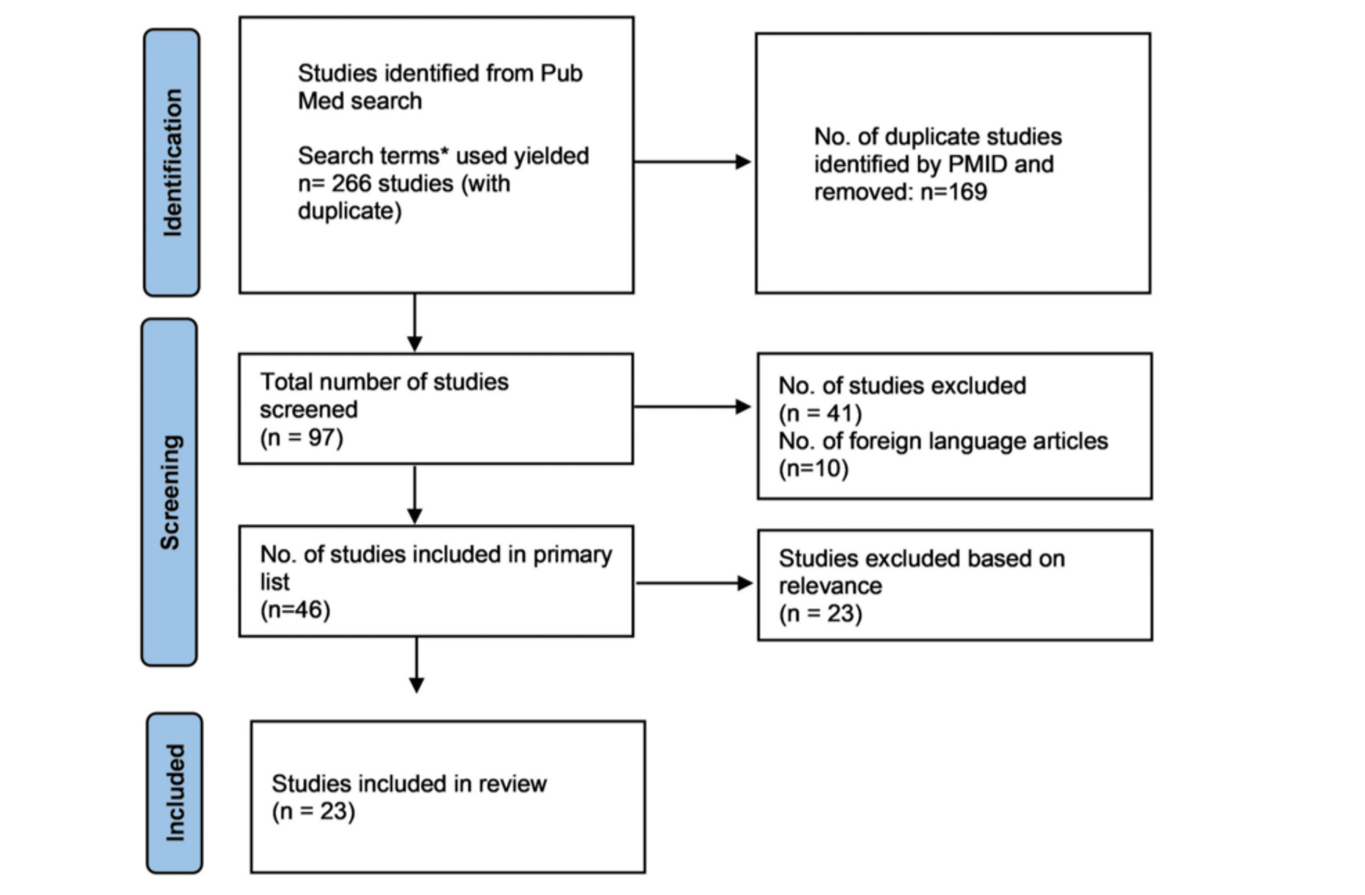
Preferred Reporting Items for Systematic Reviews and Meta-Analyses (PRISMA) flow diagram.

An extensive literature search was performed using PubMed from January 2014 to March 2024 to evaluate existing clinical evidence on silodosin in the management of LUTS associated with BPH (Figure 1). This 10-year period was selected to capture the most recent and relevant clinical data reflecting current therapeutic practices and safety profiles, considering the evolving landscape of BPH management. Keywords including “silodosin,” “benign prostatic hyperplasia,” “cardiovascular,” “efficacy,” “effectiveness,” “safety,” “adverse effect*,” and “comorbid*” were used in the search terms. The goal of this search strategy was to gather evidence regarding the role of silodosin in BPH management, especially concerning its efficacy and safety, and evidence related to pre-existing health conditions.

Inclusion Criteria

Inclusion criteria for the review were peer-reviewed clinical trials and observational studies with full-text articles in English that assessed silodosin treatment either alone or compared with placebo or other treatments, or silodosin studies on its use as a combination therapy in patients with LUTS associated with BPH. In addition, cross-referencing from identified studies ensured comprehensive coverage of relevant research.

Exclusion Criteria

Exclusion criteria were review articles, systematic literature reviews and meta-analyses, or those in which silodosin was assessed at a dose of 4 mg/day alone (i.e., the study was included if silodosin was assessed at doses of 8 mg/day or 4 mg twice a day), and studies using propiverine as a comparator.

Results

In total, 26 articles (21 clinical trials and 5 observational studies) that assessed silodosin for the treatment of LUTS associated with BPH were identified. Of these, two studies were excluded for using silodosin only at a dose of 4 mg/day, and one for using propiverine as a comparator. Consequently, 23 studies were included in this review. Details of the studies are outlined in Table 1.

**Table 1:** Studies evaluating the effectiveness/efficacy of silodosin based on efficacy measures and relevant scores. AUR: acute urinary retention; BOOI: Bladder Outlet Obstruction Index; BPH: benign prostatic hyperplasia; CT: clinical trial; IIEF: International Index of Erectile Function; IPSS: International Prostate Symptom Score; LUTS: lower urinary tract symptoms; NS: not significant; OAB: overactive bladder; OABSS: Overactive Bladder Symptom Score; PSA: prostate-specific antigen; PVR: post-void residual; Qmax: maximum urinary flow rate; QoL: quality of life; RCT: randomized controlled trial; SR: sustained release; wk: week

Author (year); study type	Patient characteristics	Treatments	IPSS total	Q_max_	PVR	QoL	OABSS
Fusco et al. (2018); [12] Single-arm, open-label, single-center, post-marketing interventional CT	• Caucasian (European) patients with severe LUTS • Age ≥50 years • IPSS ≥13 • IPSS-QoL ≥3 • prostate volume ≥30 mL • Q_max_ <15 mL/s • BOOI >40	• Silodosin 8 mg/day (N = 34) for 8 wks	Baseline: 21.6 ± 3.1 vs. 8 wks: 10.9 ± 2.2; p < 0.0001	Baseline: 7.8 ± 3.1 mL/s vs. 8 wks: 9.5 ± 3.8 mL/s; p = 0.015	Baseline: 71.1 ± 33.1 mL vs. 8 wks: 52.5 ± 23.2 mL; p < 0.0001	Baseline: 4.6 ± 0.8 vs. 8 wks: 1.5 ± 0.7; p < 0.0001	-
Cho et al. (2018); [13] Single-arm, open-label, multicenter, prospective CT	• South Korean BPH patients • Nocturia ≥2/night • Age ≥50 years • IPSS ≥8 • QoL ≥3	• Silodosin 8 mg/day (N = 112) for 12 wks	-7.93 ± 6.75; p < 0.0001 vs. baseline	-	-	-1.26 ± 1.29; p < 0.0001 vs. baseline	-2.34 ± 2.76; p < 0.0001 vs. baseline
Montorsi et al. (2016); [14] International, multicenter, open-label, single-arm, phase IV study	• European LUTS/BPH patients • Age ≥60 years • IPSS ≥12	• Silodosin 8 mg/day (N = 1,036) for 24 wks	Baseline: 18.9 ± 4.9 vs. study end: 10.6 ± 6.0; p < 0.001	-	-	Baseline: 4.0 ± 1.2 vs. study end: 2.2 ± 1.4; p < 0.001	-
Choo et al. (2014); [15] Double-blind, multicenter, prospective RCT	• Korean patients with LUTS or BPH • Age ≥ 50 years • IPSS ≥ 8 • QoL ≥ 3 • Prostate volume ≥ 20 mL • Q_max_ <15 mL/s	• Silodosin 8 mg once daily; QD (N = 215) • Silodosin 4 mg twice daily; BID (N = 209) for 12 wks	QD: -6.70 vs. BID: -6.94; NS	QD: 3.55 ± 5.93 mL/s vs. BID: 3.74 ± 6.79 mL/s; p = 0.768	QD: -10.41 ± 41.24 vs. BID: -1.33 ± 56.15; p = 0.064	QD: -1.19 ± 1.49 vs. BID: -1.40 ± 1.42; p = 0.136	-
Novara et al. (2015); [16] Pooled analysis of three RCTs - multicenter, double-blind, placebo-controlled phase III studies	• Moderate-to-severe LUTS • 1 European and 2 American studies • Age ≥ 50 years • IPSS ≥ 13 • Q_max_ 4-15 mL/s	• Silodosin 8 mg/day (N = 847) • Placebo (N = 647) for 12 wks	Significant improvements with silodosin vs. placebo when stratified by age (p < 0.001), baseline IPSS (p < 0.001), and baseline Q_max_ (p < 0.001)	Significant improvements with silodosin vs. placebo when stratified by age (p < 0.05), baseline IPSS >20 (p = 0.0006), and baseline Q_max _≤ 10 mL/s (p ≤ 0.0008)	-	Significant improvements with silodosin vs. placebo when stratified by age (p < 0.001), baseline IPSS (p < 0.002), and baseline Q_max_ (p < 0.02)	-
Eisenhardt et al. (2014); [17] Pooled analysis of three RCTs - multicenter, double-blind, placebo-controlled phase III studies	• LUTS in patients with signs or symptoms of BPH • 1 European and 2 American studies • Age ≥ 50 years • IPSS ≥ 13 • Q_max_ 4-15 mL/s • ≥2 nocturnal voids at baseline	• Silodosin 8 mg/day (N = 714) • Placebo (N = 552) for 12 wks	-	-	-	-	-
Matsumoto et al. (2020); [18] Non-blinded, open-label, single-center, prospective RCT	• Japanese patients with LUTS/BPH • Age ≥50 years • IPSS ≥8 • QoL ≥2	• Tamsulosin 0.2 mg/day (N = 22) • Silodosin 8 mg/day – 4 mg twice daily (N = 20) for 4 wks	Tamsulosin: at baseline: 14.78 ± 7.25 vs. 4 wks 9.45 ± 7.58; p < 0.01. Silodosin: at baseline 15.15 ± 5.91 vs. 4 wks 9.25 ± 4.51; p < 0.01	-	-	Tamsulosin: at baseline 4.09 ± 0.92 vs. 4 wks 2.86 ± 1.13; p < 0.01. Silodosin: at baseline 4.50 ± 1.24 vs. 4 wks 3.35 ± 1.53; p < 0.05	-
Pande et al. (2014); [19] Single-blind, parallel group RCT	• Indian BPH patients with bothersome LUTS • Age >50 years • IPSS >7	• Silodosin 8 mg/day (N = 26) • Tamsulosin 0.4 mg/day (N = 27) for 12 wks	Silodosin: Baseline 18.4 ± 3.32 vs. 12 wks 6.7 ± 2.78; p < 0.001. Tamsulosin: Baseline 18.4 ± 3.94 vs. 12 wks 7.4 ± 2.82; p < 0.001. Inter-group difference; p = 0.953	Silodosin: Baseline 15.5 vs. 12 wks 15.8; p = 0.924. Tamsulosin: Baseline 15.9 vs. 12 wks 16.4; p = 0.114. Inter-group difference; p = 0.108	Silodosin: Baseline 49.2 ± 50.38 vs. 12 wks 79.6 ± 98.60; p = 0.120. Tamsulosin: Baseline 58.9 ± 65.64 vs. 12 wks 47.4 ± 74.40; p = 0.270. Inter-group difference; p = 0.106	-	-
Manjunatha et al. (2016); [20] Comparative open-label, prospective RCTs	• Indian LUTS/BPH patients • Age ≥45 years • IPSS ≥8 • IPSS-QoL ≥3 • Q_max_ 4–15mL/s • Voided volume >100 mL	• Silodosin 8 mg/day (N = 30) • Alfuzosin SR 10 mg/day (N = 30) • Tamsulosin 0.4 mg/day (N = 30) for 12 wks	Silodosin: -13.1 ± 5.65 vs. Alfuzosin: -16.93 ± 8.7 vs. Tamsulosin -15.6 ± 7.75. Inter-group difference; p = 0.242	Silodosin: 1.33 ± 3.0 vs. Alfuzosin: 2.57 ± 4.25 vs. Tamsulosin 2.97 ± 3.0. Inter-group difference; p = 0.170	-	Silodosin: -4.13 ± 1.25 vs. Alfuzosin: -4.27 ± 1.1 vs. Tamsulosin -3.6 ± 1.59. Inter-group difference; p = 0.375	-
Kim et al. (2014); [21] Multicenter, cross-sectional, observational study	• Korean patients with LUTS suggestive of BPH • Age ≥ 45 years • Treated with a1-blocker monotherapy IPSS ≥ 8	• Silodosin 4 mg/day or 8 mg/day (N = 383) • Doxazosin 4 mg/day or 8 mg/day (N = 182) • Alfuzosin 10 mg/day (N = 486) • Tamsulosin 0.2 mg/day or 0.4 mg/day (N = 2,149)	• Silodosin: -4.02 ± 0.77; P=0.028 • Doxazosin: -3.89 ± 0.76; p = 0.041 • Alfuzosin: -4.16 ± 0.82; p = 0.032 • Tamsulosin: -3.93 ± 0.78; p = 0.011 vs. baseline	• Silodosin: -1.22 ± 0.21; P=0.088 • Doxazosin: -1.32 ± 0.28; p = 0.145 • Alfuzosin: -1.01 ± 0.93; p = 0.092 • Tamsulosin: -0.83 ± 0.47; p = 0.242 vs. baseline	• Silodosin: -1.88 ± 1.23; P=0.352 • Doxazosin: -3.52 ± 2.11; p = 0.421 • Alfuzosin: -2.35 ± 1.24; p = 0.568 • Tamsulosin: -0.78 ± 0.69; p = 0.785 vs. baseline	• Silodosin: -0.43 ± 0.37; P=0.454 • Doxazosin: -0.35 ± 0.13; p = 0.340 • Alfuzosin: -0.23 ± 0.21; p = 0.673 • Tamsulosin: -0.24 ± 0.15; p = 0.338 vs. baseline	-
Matsukawa et al. (2017); [22] Multicenter, prospective RCT	• Japanese OAB/BPH patients • storage/voiding LUTS > 3 months • Age ≥50 years • IPSS ≥8 • IPSS-QoL ≥3 • OABSS ≥3 • urinary urgency episodes ≥1/wk • prostate volume ≥20 mL • Q_max_ <15 mL/s • Residual urine <150 mL	• Silodosin 4 mg/day for 4 wks + 8 mg/day for 8 wks (N = 157) • Naftopidil 50 mg/day for 4 wks + 75 mg/day for 8 wks (N = 157)	• Silodosin: -7.5; p < 0.001 vs. baseline • Naftopidil: -6.2; p < 0.001 vs. baseline. Inter-group difference; p = 0.08	• Silodosin: +3.6 mL/s; p < 0.001 vs. baseline • Naftopidil: +2.1 mL/s; p < 0.001 vs. baseline. Inter-group difference; p = 0.01	• Silodosin: -17 mL; p < 0.001 vs. baseline • Naftopidil: -13 mL; p = 0.002 vs. baseline. Inter-group difference; p = 0.05	• Silodosin: -1.9; p < 0.001 vs. baseline • Naftopidil: -1.6; p < 0.001 vs. baseline. Inter-group difference; p = 0.005	• Silodosin: -2.8; p < 0.001 vs. baseline •Naftopidil: -2.3; p < 0.001 vs. baseline. Inter-group difference; p = 0.03
Matsukawa et al. (2019); [23] Post-hoc analysis of a multi-center prospective RCT	• Japanese patients with BPH/OAB • Age ≥50 years • IPSS ≥8 • IPSS-QoL ≥3 • OABSS ≥3 • urinary urgency episodes ≥1/wk • prostate volume ≥20 mL • Q_max_ <15 mL/s • Residual urine <150 mL • Storage/voiding LUTS >3 months	• Silodosin 8 mg/day (N = 157) • Naftopidil 75 mg/day (N=157) for 12 wks	-	-	-	-	OABSS urgency subscore <2, indicating a good response. Silodosin: 68.2% vs. Naftopidil: 31.8%; p < 0.001
Masciovecchio et al. (2017); [24] Open-label, single-center, prospective CT	• Italian LUTS/BPH patients previously treated with tamsulosin (0.4 mg/day) for ≥12 months • IPSS ≥8 • IPSS-QoL ≥3 • prostate volume ≤40 mL • Q_max_ <15 mL/s • PVR ≤150 mL • PSA <4 ng/mL	• Silodosin 8 mg/day (N = 96) for 8 wks	Baseline: 20.0 ± 4.4 vs. 8 wks: 18.6 ± 4.5; p < 0.03	Baseline: 11.9 ± 1.5 mL/s vs. 8 wks: 12.0 ± 1.7 mL/s; NS	Baseline: 82.3 ± 28.3 mL vs. 8 wks: 77.2 ± 27.1 mL; NS	Change from baseline: -0.8 ± 1.0; p < 0.001	-
Osman et al. (2015); [25] Prospective, multicenter, open-label extension study	• European patients who completed the 12-wk double-blind study with silodosin 8 mg/day, tamsulosin 0.4 mg/day, or placebo	• Silodosin 8 mg/day (N = 500) for 9 months	Previously received placebo: -2.7 ± 3.8 vs. silodosin: -0.82 ± 4.2 vs. tamsulosin: -0.83 ± 3.8	Previously received placebo: +0.47 ± 5.7 mL/s vs. silodosin: +0.63 ± 4.9 mL/s vs. tamsulosin: +0.01 ± 4.7 mL/s	-	Previously received placebo: -0.72 ± 1.0 vs. silodosin: -0.41 ± 1.0 vs. tamsulosin: -0.31 ± 1.1	-
Takahashi et al. (2019); [26] Post-marketing observational study	• Japanese BPH patients • received α1-blocker other than silodosin for ≥3 months but had experienced unsatisfactory treatment outcomes • QoL >2	• Silodosin dose not provided (N = 3,144 for effectiveness) for 3 months	All: -4.1 ± 6.0: p < 0.0001 vs. Previously used tamsulosin: -4.2 ± 6.2; p < 0.0001 vs. Previously used naftopidil: -4.1 ± 5.7; p < 0.0001	-	All: -17.2 ± 53.9: p < 0.0001 vs. Previously used tamsulosin: -17.5 ± 55.6; p < 0.0001 vs. Previously used naftopidil: -16.8 ± 52.0; p < 0.0001	All: -1.3 ± 1.3: p < 0.0001 vs. Previously used tamsulosin: -1.3 ± 1.4 p < 0.0001 vs. Previously used naftopidil: -1.2 ± 1.3; p < 0.0001	All: -1.1 ± 2.3: p < 0.0001 vs. Previously used tamsulosin: -1.2 ± 2.4 p < 0.0001 vs. Previously used naftopidil: -1.1 ± 2.1; p < 0.0001
Ichihara et al. (2018); [27] Multicenter, non-randomized, prospective observational study	• Japanese participants who had LUTS associated with BPH • α-blocker other than silodosin for ≥8 wks • Age ≥ 50 years • IPSS ≥ 13 • QoL ≥ 4	• Silodosin 8 mg/day (4 mg twice daily; N = 73) for 8 wks	Baseline: 19.1 ± 5.3 vs. 8-wks: 14.1 ± 6.4; p < 0.001	-	-	Baseline: 4.4 ± 0.6 vs. 8 wks: 3.6 ± 1.2; p < 0.001	Baseline: 5.8 ± 3.0 vs. 8 wks: 4.9 ± 2.8; p = 0.009
AbdelRazek et al. (2022); [28] Prospective RCT	• Egyptian patients with LUTS associated with BPH • Age >50 years • IPSS >8 • IIEF score >12 • Q_max_ <10 mL/s • PVR urine <200 cc	• Tadalafil 5 mg/day (N = 101) • Silodosin 8 mg/day (N = 102) • Combination (N = 105) for 3 months	Tadalafil alone: 17.6 vs. Silodosin alone: 16.7 vs. Combination therapy: 15.6 • Tadalafil vs. silodosin; p ≤ 0.003 • Tadalafil vs. combination; p < 0.001 • Silodosin vs. combination; p < 0.001	Tadalafil alone: 7.2 ± 2.0 mL/s vs. Silodosin alone: 8.1 ± 2.3 mL/s vs. Combination therapy: 8.7 ± 2.0 mL/s • Tadalafil vs. silodosin; p = 0.002 • Tadalafil vs. combination; p < 0.001 • Silodosin vs. combination; p = 0.069	Tadalafil alone: 11.1 ± 7.1 mL vs. Silodosin alone: 13.3 ± 6.4 mL vs. Combination therapy: 15.5 ± 6.4 mL • Tadalafil vs. silodosin; p = 0.02 • Tadalafil vs. combination; p < 0.001 • Silodosin vs. combination; p = 0.014	-	-
Yoshida et al. (2017); [29] Non-blinded, parallel-group, multicenter, prospective RCT	• Japanese LUTS/BPH patients • diagnosed based on ultrasound or digital rectal examination • Age ≥60 years • IPSS ≥13 • IPSS-QoL ≥3 • prostate volume ≥20 mL • Residual urine <100 mL	Period 1 • Silodosin 8 mg/day – 4 mg twice daily (N = 93) • Tadalafil 5 mg/day (N = 95) for 8 wks. Period 2 Tadalafil group received either Silodosin (N = 47) or Tadalafil (N = 42) for another 8 wks	Period 1: Silodosin: -10.1 ± 6.4; p < 0.0001 vs. baseline Tadalafil: -8.0 ± 6.3; p < 0.0001 vs. baseline. Inter-group difference; p = 0.0277. Period 2: no intergroup difference; p = 0.8787	Period 1: Significant improvement only with silodosin (2.04 ± 7.30 mL/s; p = 0.0146). Inter-group difference; p = 0.0571. Period 2: no intergroup difference; p = 0.8924	Period 1: No significant improvement with silodosin (p = 0.4796) or tadalafil (p = 0.4785). Inter-group difference; p = 0.8753. Period 2: no intergroup difference; p = 0.1439	Period 1: Silodosin: -1.5 ± 1.4; p < 0.0001 vs. baseline Tadalafil: -0.9 ± 1.3; p < 0.0001 vs. baseline. Inter-group difference; p = 0.0032. Period 2: no intergroup difference; p = 0.3746	Period 1: Silodosin: -3.2 ± 2.7; p < 0.0001 vs. baseline Tadalafil: -2.2 ± 2,6; p < 0.0001 vs. baseline Inter-group difference; p = 0.0117 Period 2: no intergroup difference; p = 0.5021
Cakiroglu et al. (2017); [30] Prospective observational study	• Turkish patients having LUTS due to BPH with ejaculatory concerns • mild-moderate LUTS, seeking preservation of fertility • Age ≤ 60 years • Prostate volume ≤ 40 cc	• TUIP (N = 96) • Silodosin 8 mg/day (N = 96) for 12 months	TUIP: 4.7 ± 2.0 vs. Silodosin: 5.7 ± 2.6; p = 0.005	TUIP: 19.6 ± 3.9 mL/s vs. Silodosin: 15.0 ± 4.7 mL/s; p = 0.000	TUIP: 20.8 ± 23.2 mL/s vs. Silodosin: 26.5 ± 26.8 mL/s; p = 0.123	-	-
Abuelnaga et al. (2024); [31] Prospective RCT	• Egyptian patients with the first episode of AUR secondary to BPH • no previous use of α-blockers	N = 260 catheterized • Silodosin 8 mg/day 3 days (N = 130) • Silodosin 8 mg/day 7 days (N = 130) Followed by catheter removal	-	3-day group: 12.66 ± 2.49 mL/s vs. 7-day group: 12.39 ± 3.41 mL/s; p = 0.578	3-day group: 73.9 ± 46.03 mL vs 7-day group: 58.11 ± 54.6 mL; p = 0.52	-	-
Matsukawa et al. (2019); [32] Open-label, single-center RCT	• Japanese patients with persistent OAB symptoms post-silodosin monotherapy (8 mg/day for 12–24 wks) • Age ≥50 years • IPSS ≥8 • IPSS-QoL ≥3 • OABSS ≥3 • urinary urgency episode ≥1/wk • prostate volume ≥25 mL • Q_max_ <15 mL/s • Residual urine <150 mL	• Fesoterodine 4 mg/day + Silodosin 8 mg/day (N = 50) • Mirabegron 50 mg/day + Silodosin 8 mg/day (N = 52)	Silodosin + Feso: -4.1 ± 4.1; p < 0.001 vs. baseline Silodosin + Mira: -3.8 ± 4.0; p < 0.001 vs. baseline. Inter-group difference; p = 0.68	Silodosin + Feso: 1.1 ± 3.7; p = 0.07 vs. baseline Silodosin + Mira: 1.4 ± 2.6; p = 0.02 vs. baseline. Inter-group difference; p = 0.66	Silodosin + Feso: 16 ± 43; p = 0.03 vs. baseline Silodosin + Mira: -3 ± 43; P=0.69 vs. baseline. Inter-group difference; p = 0.02	Silodosin + Feso: -1.5 ± 1.1; p < 0.001 vs. baseline Silodosin + Mira: -1.1 ± 1.0; p < 0.001 vs. baseline. Inter-group difference; p = 0.04	Total OABSS Silodosin + Feso: -2.8 ± 2.1; p < 0.001 vs. baseline Silodosin + Mira: -1.5 ± 2.2; p = 0.004 vs. baseline. Inter-group difference; p = 0.004
Matsukawa et al. (2017); [33] Single-center, prospective RCT	• Japanese LUTS/BPH patients • Age ≥45 years • IPSS ≥8 • IPSS-QoL ≥3 • prostate volume ≥30 mL • Q_max_ <15 mL/s • Residual urine <150 mL	After 12 months of silodosin + dutasteride • Silodosin 8 mg/day + Dutasteride 0.5 mg/day (N = 57) • Dutasteride 0.5 mg/day (N = 60) for an additional 12 months	Silodosin + Dutasteride: -7.0; p < 0.001 vs. baseline Dutasteride: -7.0; p < 0.001 vs. baseline. Inter-group difference; p = 0.79	Silodosin + Dutasteride: +2.4 mL/s; p < 0.001 vs. baseline Dutasteride: +2.1 mL/s; p = 0.002 vs. baseline. Inter-group difference; p = 0.83	Silodosin + Dutasteride: -37 mL; p < 0.001 vs. baseline Dutasteride: -26 mL; p = 0.01 vs. baseline. Inter-group difference; p = 0.49	Silodosin + Dutasteride: -1.8; p < 0.001 vs. baseline Dutasteride: -1.9; p < 0.001 vs. baseline. Inter-group difference; p = 0.66	Silodosin + Dutasteride: -1.8; p < 0.001 vs. baseline Dutasteride: -1.8; p < 0.001 vs. baseline. Inter-group difference; p = 0.83
Hagiwara et al. (2016); [34] Prospective, single-arm observational study	• Japanese patients with the first episode of spontaneous AUR	• Bladder decompression with indwelling urinary catheter + Silodosin 8 mg/day + Dutasteride 0.5 mg/day + TWOC every 2 wks until 12 wks (N = 80)	Baseline: 26 vs. 2 wks: 8; p < 0.001. Sustained improvement through 12 wks	Gradual increase at 2, 4, 8, and 12 wks (p < 0.001 vs. baseline). Exact values not provided	Significant decrease at 2, 4, 8, and 12 wks (p < 0.001 vs. baseline). Exact values not provided	Gradual decrease at 2, 4, 8, and 12 wks (p < 0.001 vs. baseline). Exact values not provided	-

Efficacy/Effectiveness

Silodosin alone: The efficacy of silodosin alone (i.e., no comparator) in treating LUTS associated with BPH and related conditions was investigated in four clinical trials. The results were consistent across studies, indicating significant improvements across a range of urological outcomes, including International Prostate Symptom Score (IPSS), maximum urinary flow rate (Qmax), post-void residual volume (PVR), QoL, International Index of Erectile Function (IIEF), and overactive bladder (OAB) symptom scores (OABSS). A summary of specific study-related outcomes is outlined in Table 1.

Fusco et al. (2018) [12] investigated the impact of silodosin (8 mg/day for eight weeks) on obstructive urodynamic parameters in patients with LUTS associated with BPH (N = 34). In addition to significant improvements in IPSS total score and storage, voiding, and QoL sub-scores (all p < 0.0001), a significant reduction was observed in the mean bladder outlet obstruction index (BOOI) from 70.6 ± 18.9 to 39.2 ± 18.3 (p < 0.0001). Additionally, detrusor opening pressure decreased significantly (p = 0.0031), and improvements in Qmax (p = 0.015) and bladder contractility index (p = 0.0274) were observed. Cho et al. (2018) [13] examined the efficacy of silodosin (8 mg/day for 12 weeks) in reducing nocturia in patients with BPH (N = 112). A significant reduction was observed in nocturia episodes by -1.12 ± 1.05 events (p < 0.0001), with 65.2% of patients experiencing ≥25% reduction in nocturia episodes. Furthermore, 63.4% of patients showed a significant decrease in IPSS scores, with an overall improvement in total IPSS score by -7.93 ± 6.75 (p < 0.0001). The IPSS voiding and storage sub-scores and other efficacy endpoints, such as OABSS and nocturia score, also showed significant improvements (p < 0.0001). Another study treating BPH patients with associated LUTS (N = 1,036) with silodosin (8 mg/day for 24 weeks) showed that its efficacy, including improvements in IPSS, nocturia, and QoL, was sustained up to the six-month study period [14]. A randomized controlled trial (RCT) by Choo et al. (2014) [15] comparing the administration of silodosin 8 mg once-daily versus 4 mg twice-daily in patients with LUTS associated with BPH (N = 424) found no significant differences in improvements in efficacy between the groups.

Silodosin versus comparators: Nineteen studies examined the efficacy of silodosin compared with placebo (n = 2) or various pharmacological treatments (n = 17) for LUTS associated with BPH, including α1-blockers, phosphodiesterase inhibitors, 5α-reductase inhibitors, and combination therapies.

Silodosin versus placebo: Two studies reporting a pooled analysis of three RCTs found that when compared with placebo [16,17], silodosin (8 mg/day for 12 weeks) significantly improved IPSS-related parameters irrespective of age, baseline IPSS, and baseline Qmax [16], as well as nocturia in patients with LUTS associated with BPH [17].

Silodosin versus other α1-blockers: Several studies compared the efficacy/effectiveness of silodosin with other α1-blockers such as tamsulosin, naftopidil, and alfuzosin for the treatment of LUTS associated with BPH. Matsumoto et al. (2020) [18] found that both silodosin (4 mg twice daily; N = 20) and tamsulosin (0.2 mg/d; N = 22) significantly improved IPSS and QoL scores over four weeks of treatment (p < 0.05). Only silodosin demonstrated significant constipation relief (p < 0.05) and improvement of stool form (p < 0.05) when compared with baseline. Another RCT comparing a higher dose of tamsulosin (0.4 mg/day; N = 27) versus silodosin (8 mg/day; N = 26) for 12 weeks found the IPSS score, PVR, and uroflowmetry parameters (e.g., Qmax, voiding volume, flow time) to be comparable between the groups [19]. The IPSS sexual function score did not change with tamsulosin; however, it worsened with silodosin (baseline: 0 vs. study end: 1.5; p < 0.001). An RCT comparing silodosin (8 mg/day; N = 30), tamsulosin (0.4 mg/day; N = 30), and sustained-release alfuzosin (10 mg/day; N = 30) showed significant improvement in IPSS and QoL among all three groups after 12 weeks of treatment; however, no significant intergroup differences in total IPSS, QoL, and Qmax were observed [20]. Of note, Kim et al. (2014) [21] examined the frequency and reason(s) for prescription change of four different α1-blockers, namely, silodosin (N = 383), tamsulosin (N = 2,149), alfuzosin (N = 486), and doxazosin (N = 182) in patients with LUTS associated with BPH. The proportion of prescription change was the lowest for the silodosin group (16.3% vs. 20.2% tamsulosin; 25.5% alfuzosin; 25.8% doxazosin; p < 0.05). Among those who changed prescriptions, lack of efficacy was the most common reason across all treatment groups. While significant improvement was observed in the IPSS total score (p < 0.05), the improvements in Qmax, PVR, and QoL were not significant across all four treatments.

In the SNIPER study, Matsukawa et al. (2017) [22] reported that silodosin (4 mg/day for four weeks + 8 mg/day for eight weeks; N = 157) resulted in superior IPSS-QoL and OABSS at 12 weeks (p < 0.01), greater Qmax (p = 0.01) and lower PVR (p = 0.05) compared with naftopidil (50 mg/day for four weeks + 75 mg/day for eight weeks; N = 157), suggesting that silodosin is particularly effective in BPH patients with OAB symptoms. A post-hoc analysis of the study identified that larger PVR and having urgency urinary incontinence before treatment initiation are key pre-treatment factors associated with insufficient improvement of OAB symptoms with an α1-blocker [23]. Additionally, the use of silodosin resulted in greater improvement of storage symptoms when compared with naftopidil.

Four studies assessed the efficacy of silodosin treatment after switching from other α1-blockers. Masciovecchio et al. (2017) [24] showed that patients who did not respond to tamsulosin (0.4 mg/day) for ≥12 months exhibited significant improvement in total IPSS (-1.3 ± 1.4; p < 0.03) and QoL (-0.8 ± 1.0; p < 0.001) after switching to silodosin (8 mg/day) in just eight weeks. However, there were no substantial changes in voiding symptoms, Qmax, and PVR. A nine-month extension study with silodosin (8 mg/day; N = 500) showed that patients who were previously treated for 12 weeks with placebo showed a greater reduction in IPSS (-2.7 ± 3.8) compared to those who previously received silodosin (8 mg/day; -0.82 ± 4.2) or tamsulosin (0.4 mg/day; -0.83 ± 3.8) [25]. Similar improvements were observed for QoL; however, changes in Qmax were marginal. In an observational study, patients non-responsive to α1-blockers (N = 3,144) such as tamsulosin (53.6%) or naftopidil (45.5%) for ≥3 months, demonstrated significant improvements in IPSS, QoL, OABSS, and residual urine volume after switching to silodosin for three months (p < 0.0001); however, the dose of silodosin was not mentioned [26]. Similar results were observed in another observational study that assessed the efficacy of silodosin (8 mg/day for eight weeks; N = 73) in BPH patients with mild LUTS who responded poorly to a previously prescribed α1-blocker (tamsulosin or naftopidil) for a mean duration of 30.3 months [27].

Silodosin versus tadalafil: The efficacy of silodosin (8 mg/day; N = 102) was compared with tadalafil (5 mg/day; N = 101), a phosphodiesterase type-5 inhibitor, in a study by AbdelRazek et al. (2022) [28]. Three months post-treatment, silodosin demonstrated significant improvement in mean IPSS (p ≤ 0.003), Qmax (p = 0.002), International Index of Erectile Function (IIEF) score (p < 0.001), and PVR (p = 0.02) when compared with tadalafil. Similarly, an RCT by Yoshida et al. (2017) [29] demonstrated that while both silodosin (8 mg/day) and tadalafil (5 mg/day) improved IPSS after eight weeks of treatment (Period 1), the improvement was significantly greater in patients treated with silodosin (-10.1 ± 6.4 vs. -8.0 ± 6.3 for tadalafil; p = 0.0277). Patients treated with tadalafil switched to silodosin or continued tadalafil for an additional eight weeks (Period 2). During Period 2, both silodosin and tadalafil demonstrated further improvements in IPSS total score, QoL, and OABSS. Although there were no statistically significant intergroup differences observed for any of these parameters, those who switched to silodosin demonstrated a faster onset of improvements when compared with those who continued tadalafil.

Silodosin versus transurethral incision of the prostate: The efficacy of silodosin (8 mg/day; N = 96) vs. transurethral incision of the prostate (TUIP) (N = 96) in patients with bladder outlet obstruction due to small prostate size (volume ≤40 mL) was assessed by Cakiroglu et al. (2017) [30]. After 12 months of treatment, both groups showed significant improvements in IPSS, Qmax, and PVR, with the TUIP group showing significantly greater improvements. There were no significant intergroup differences in the IIEF scores post-treatment (p = 0.389).

Silodosin used in combination with other therapies: While the above studies consistently demonstrated the efficacy/effectiveness of silodosin as a monotherapy, combination therapy studies indicate that its efficacy is often enhanced when combined with other agents. Five studies examined the comparative efficacy/effectiveness of silodosin monotherapy versus combination therapies for the treatment of LUTS associated with BPH, OAB, and sexual dysfunction.

Abuelnaga et al. (2024) [31] compared early (three-day; N = 130) versus late (seven-day; N = 130) catheter removal in patients with acute urinary retention who were simultaneously treated with silodosin (8 mg/day). The success rate of the trial without a catheter (TWOC) was higher in the seven-day group (68%) versus the three-day group (57%), although this difference was not statistically significant (p = 0.096). AbdelRazek et al. (2022) [28] compared the efficacy of silodosin (8 mg/day; N = 102), tadalafil (5 mg/day; N = 101), and their combination (N = 105) for treating LUTS associated with BPH. Change in Qmax improved significantly in the combination therapy group versus tadalafil alone (-1.45; p < 0.001); however, no difference was observed with silodosin alone (-0.53; p = 0.069). Changes in IPSS and PVR were significantly better in the combination group versus either monotherapy groups.

The efficacy of silodosin in combination with either mirabegron (β3-agonist; 50 mg/day) or fesoterodine (anticholinergic agent; 4 mg/day) for managing OAB symptoms that persisted even with 12-24 weeks of silodosin treatment (8 mg/day) was investigated by Matsukawa et al. (2019) [32]. Although both groups demonstrated significant improvements following 12 weeks of treatment, silodosin + fesoterodine showed greater improvement in OABSS (p = 0.004), IPSS-QoL (p = 0.04), and alleviation of detrusor overactivity (p = 0.03) compared with silodosin + mirabegron. Moreover, PVR significantly increased following treatment with silodosin + fesoterodine (p = 0.03).

In patients with LUTS associated with BPH treated with a combination therapy of silodosin (8 mg/day) and dutasteride (0.5 mg/day), a 5α-reductase inhibitor, for 12 months, Matsukawa et al. (2017) [33] compared the effects of continuing the combination therapy (N = 57) versus switching to dutasteride alone (i.e., following silodosin withdrawal; N = 60) for an additional 12 months. No significant differences in IPSS, OABSS, and other voiding parameters were observed between the two groups. Regarding storage function, additional improvements were observed in bladder capacity and detrusor overactivity in the combination group, whereas these parameters worsened upon withdrawal of silodosin in the dutasteride alone group. IPSS and related parameters worsened after re-randomization in 26.3% and 38.3% of patients in the combination and dutasteride alone groups, respectively; body weight and body mass index were found to be higher in these patients. Therefore, the withdrawal of silodosin must be done with careful consideration in patients with a high body mass index. Hagiwara et al. (2016) [34] assessed the combination therapy of silodosin (8 mg/day) and dutasteride (0.5 mg/day) in addition to bladder decompression in BPH patients with acute urinary retention (N = 80). After 12 weeks of treatment, the success rate of TWOC was 88.8%, and significant improvements were observed in IPSS, IPSS-QoL, voided volume, PVR, and Qmax versus baseline (p < 0.001).

Efficacy/Effectiveness of Silodosin on Nocturia

Frequent nighttime urination is a symptom that often bothers BPH patients, because of which they have difficulty sleeping, ultimately negatively impacting QoL [17]. Five studies assessed the effect of silodosin on various nocturia-related parameters.

The open-label study by Cho et al. (2018) [13] included patients with nocturia (≥2 events/night). After 12 weeks of treatment with silodosin, nocturia events decreased significantly (-1.12 ± 1.05; p < 0.0001), the International Consultation on Incontinence Questionnaire-Nocturia score significantly improved (p < 0.0001), and 65.18% of patients demonstrated ≥25% reduction in nocturia rate. Eisenhardt et al. (2014) [17] pooled results from three RCTs and showed that the proportion of patients with improvements in nocturia was significantly higher with silodosin (53.4% vs. 42.8% with placebo; p < 0.0001) and fewer patients reported worsening of nocturia following treatment (9.0% vs. 14.3% with placebo; p < 0.0001). Nocturnal voiding frequency decreased significantly with silodosin (-0.8 vs. -0.6 with placebo; p < 0.001), and a higher percentage of individuals attained ≥1 reduction in nocturnal voiding episodes within 12 weeks (silodosin: 61% vs. placebo: 49%; p = 0.0003).

The effect of silodosin on nocturia was compared with other treatments in three RCTs. Both silodosin and naftopidil significantly improved the IPSS-nocturia sub-score after 4 and 12 weeks of treatment (p < 0.001); however, there was no significant difference between groups [22]. When compared with tadalafil, silodosin showed a significantly greater improvement in IPSS-nocturia sub-score (p = 0.0387) and OABSS-nocturia sub-score (p = 0.0064) after eight weeks [29]. Both groups, patients who continued tadalafil or switched to silodosin, showed additional improvements in the OABSS-nocturia sub-score; however, the inter-group difference was not significant. In the study by Manjunatha et al. (2016) [20], the percentage improvement from baseline in the IPSS-nocturia sub-score was the greatest with alfuzosin (60.32%), followed by silodosin (49.01%) and tamsulosin (42.43%).

Efficacy/Effectiveness in Benign Prostatic Hyperplasia Patients With Comorbidities

In all studies (except one) [30], the mean age of patients was >60 years. These patients are likely to have one or more comorbidities, given that the prevalence of comorbidities increases in the elderly [35]. Several studies excluded patients with severe comorbidities associated with major organs, such as cardiac disease and renal disease; however, some studies assessed the performance of silodosin in BPH patients with comorbidities.

Montorsi et al. (2016) [14], in a phase IV, multicenter, open-label study, found silodosin to be effective in BPH patients with comorbidities. The study consisted of 1,036 patients, with a mean age of 67.8 ± 5.7 years, and 56.0% were taking antihypertensive medication, 45.6% had cardiovascular disease, 2.4% had diabetes, while 15.1% had both cardiovascular disease and diabetes. Silodosin had significant improvements in LUTS; 77.1% of all patients demonstrated a ≥25% decrease in IPSS, and 80.8% showed an overall decrease of >3 points in their total IPSS score compared to baseline. A mean change of -8.3 ± 6.1 on the total IPSS was recorded (p < 0.0001), with significant reductions in the IPSS sub-scores for storage, voiding, and QoL (p < 0.0001). Importantly, silodosin efficacy was comparable when stratified by age, concomitant cardiovascular disease and diabetes, and the use of antihypertensives. Osman et al. (2015) [25] demonstrated further evidence of the effectiveness of silodosin in patients with comorbidities in an open-label extension study (N = 185). In this study, 42% of patients had arterial hypertension, and most were on cardiovascular medications. The study found marked reductions in IPSS, as well as in storage and voiding sub-scores, and improved QoL, particularly in de novo silodosin patients; however, no formal statistical analysis was performed. Similarly, other studies that demonstrated that silodosin was efficacious/effective at improving LUTS showed similar improvements when stratified by age [16,26], body mass index [26], and those taking antihypertensives or with renal impairment [16]. In addition, Kim et al. (2014) [21] identified that none of the patients treated with silodosin changed their prescription due to cardiovascular comorbidity, as opposed to 9%, 5%, and 2% in the doxazosin, alfuzosin, and tamsulosin groups, respectively.

Overall Safety

The safety of silodosin was evaluated across all studies (except two) (Table 2).

**Table 2:** Studies evaluating the safety of silodosin in BPH patients. BPH: benign prostatic hyperplasia; AE: adverse effect

Parameter	Number of studies	Summary of findings	References
AEs	21	Silodosin was generally well-tolerated with mild AEs. Common AEs included retrograde ejaculation and ejaculatory disorders	[12-16,18-22,24-34]
Retrograde ejaculation or ejaculatory disorder	14	Noted as a frequent side effect. Incidence across studies ranged from 0.85% to 34.4% in patients treated with silodosin	[12-16,20,22,24-26,28-30,33]
Cardiovascular safety	7	Silodosin was not associated with QTc prolongation. The incidence of dizziness and hypotension was low with silodosin. One study found no change in blood pressure and heart rate with silodosin over 9 months of treatment. Overall, no significant cardiovascular issues were reported, and a favorable safety profile was maintained	[14,15,20,25,26,28,29]
Gastrointestinal symptoms	8	Silodosin was associated with gastrointestinal AEs such as diarrhea and dry mouth; however, the incidence was low. One study reported a serious case of intestinal obstruction with silodosin which led to study discontinuation	[13,14,16,23,25-27,29]
Discontinuation due to AEs	10	Discontinuation rates due to AEs were generally low, with most discontinuations due to mild AEs, such as ejaculatory problems	[14-16,22,25,27-29,32,33]

Overall, silodosin was well-tolerated by patients, with most adverse events (AEs) being mild and a low incidence of discontinuation due to AEs. Among the common side effects, retrograde ejaculation or ejaculatory disorder was frequently reported with silodosin. Across studies, the proportion of silodosin-treated patients reporting retrograde ejaculation or ejaculatory disorder ranged from 0.85% to 34.4% and was higher with silodosin when compared with other treatments in some studies [13,19,20,28-30]. Interestingly, Choo et al. (2014) [15] reported that patients treated with silodosin at a dose of 4 mg twice-daily were more likely to experience retrograde ejaculation (22.12% vs. 15.09%) or ejaculation disorder (8.65% vs. 6.60%) compared with those administered 8 mg once-daily.

In terms of gastrointestinal AEs, Matsumoto et al. (2020) [18] reported that silodosin (8 mg/day for four weeks) significantly increased the Bristol Stool Form Scale, suggesting that it may cause diarrhea and loose stools more frequently than tamsulosin. Similarly, other studies reported gastrointestinal AEs such as diarrhea and dry mouth with silodosin; however, the incidence was low [13,14,16,22,25-27,29]. Yoshida et al. (2017) [29] reported a serious case of intestinal obstruction in one patient who switched from tadalafil to silodosin, leading to study discontinuation.

In the study by Abuelnaga et al. (2024) [31], although TWOC was more successful in the seven-day group versus the three-day group, catheter-related complications, such as urinary leakage, acute urinary tract infection, and hematuria, were notably higher in the seven-day group (48.5% vs. 16.2% in the three-day group; p ≤ 0.001).

Cardiovascular-related safety: Several studies report a low risk of cardiovascular-related AEs with silodosin. For instance, in the RCT by Manjunatha et al. (2016) [20], corrected QT(QTc) prolongation (>45 ms), which is associated with an increased risk of arrhythmia [36], was reported in a few patients treated with alfuzosin (n = 2) and tamsulosin (n = 3); however no cases of QTc prolongation were reported with silodosin. The incidence of dizziness and hypotension, or more specifically, orthostatic hypotension, was consistently low (≤2.1%) across studies following treatment with silodosin and comparable with other treatments [14,15,20,25,26,28,29]. Osman et al. 2015 [25] found no change in systolic and diastolic blood pressure and heart rate in patients treated with silodosin for nine months. In an RCT by Novara et al. (2015) [16], the incidence of cardiovascular AEs was comparable between silodosin- and placebo-treated groups. Furthermore, patients taking antihypertensive drugs concomitantly with silodosin did not show an increased risk of dizziness or orthostatic hypotension. Additionally, in the study by Kim et al. (2014) [21], prescription change owing to hemodynamic AEs, such as dizziness, hypotension, and headache, was significantly lower with silodosin (2.4% vs. 31.0% doxazosin, 18.9% alfuzosin, and 13.3% tamsulosin; p < 0.006).

Discussion

Silodosin has consistently been shown to be efficacious and effective in the treatment of LUTS associated with BPH, demonstrating significant improvements in symptom relief, including reductions in total IPSS, storage, voiding, nocturia episodes, and improvement in the QoL of patients. When compared with other α1-blockers, such as tamsulosin and naftopidil, silodosin offered superior symptom control. Additionally, in combination therapies with drugs such as tadalafil or dutasteride, silodosin has proven beneficial for more comprehensive symptom management. Silodosin was associated with mild AEs, with retrograde ejaculation or ejaculatory disorders being the most frequent. Notably, silodosin’s selective targeting of α-1A adrenoceptors contributes to a favorable cardiovascular safety profile, with minimal concerns regarding QTc prolongation or significant cardiovascular events. In addition, silodosin has a quick onset of action [37] and one study showed that efficacy was sustained for up to six years [38], making it the treatment of choice for patients with BPH, particularly in those with comorbidities, especially cardiovascular conditions.

Efficacy/Effectiveness and Safety of Silodosin in the Context of the Treatment Landscape for BPH in India

In India, the treatment of BPH involves a range of therapeutic options tailored to the diverse needs of patients [7]. The USI 2021 guidelines recommend α-blockers for the treatment of moderate-to-severe LUTS, irrespective of prostate volume [7]. Recent clinical studies summarized in this review indicate that silodosin significantly improves LUTS and QoL in BPH patients, demonstrating a higher success rate in symptom relief compared with that achieved by using other α-blockers such as tamsulosin and naftopidil [18,23,24,26,27].

The AUA 2021 guidelines (amended in 2023) recommend that the choice of α-blockers used for managing LUTS associated with BPH must be based on patient characteristics such as age and comorbidities [6]. For instance, tamsulosin and silodosin are not recommended in patients scheduled to undergo cataract surgery as they may cause intraoperative floppy iris syndrome [7,39,40]. Likewise, while most patients treated with silodosin reported improved erectile function [41], given that silodosin is associated with increased incidence of retrograde ejaculation or ejaculatory disorders [13,19,20,28-30], younger and sexually active men must be informed regarding these AEs, as it may impact treatment adherence [42,43]. However, the selective action of silodosin on α1A-adrenoreceptors minimizes side effects, particularly cardiovascular issues [44], which is crucial in the context of the Indian population where cardiovascular comorbidities are prevalent [45,46]. In BPH patients with comorbidities, Osman et al. (2015) [25] reported a marked reduction in total IPSS, voiding and storage sub-scores, and improvement in QoL in de novo silodosin patients (previously received placebo) following nine months of treatment. In addition, they found that silodosin could alleviate these symptoms without causing significant fluctuations in blood pressure or heart rate, an important factor given the common occurrence of hypertension among Indian patients [45]. Comparative studies showed that silodosin has a better safety profile with regard to cardiovascular and hemodynamic AEs. Notably, there is no significant evidence suggesting that silodosin causes QTc prolongation [20], a condition that can predispose patients to arrhythmias [36]. In addition, the incidence of dizziness and hypotension was low with silodosin treatment [14,15,20,25,26,28,29]. The efficacy of silodosin in alleviating LUTS and a favorable cardiovascular safety profile emphasize its use as a preferred treatment option in BPH patients with cardiovascular comorbidities.

Research suggests that while monotherapy with silodosin is effective, combination treatments can offer additional benefits in managing persistent symptoms [28,32-34]. Combination therapies, including α-blockers together with 5α-reductase inhibitors, such as dutasteride, are recommended by the USI 2021 guidelines to address moderate-to-severe LUTS, especially in men with a high risk of disease progression [7]. Similarly, the EAU 2024 guidelines [5] recommend using α1-blockers with phosphodiesterase 5 inhibitors, such as tadalafil, in patients with bothersome LUTS, in particular, those who are sexually active and would like to improve erectile function. Matsukawa et al. (2017) [33] demonstrated that patients who continued combination therapy with silodosin and dutasteride sustained improvements in symptoms associated with storage function. In contrast, the group that withdrew silodosin (i.e., continued dutasteride alone) experienced a slight worsening in symptom relief, emphasizing the potential benefits of continuing silodosin for prolonged symptom management.

Overall, the approach to BPH treatment in India incorporates both monotherapy and combination strategies, with silodosin being a key option due to its effectiveness and favorable cardiovascular safety profile.

Strengths and limitations

This narrative review draws upon data from a wide range of recent studies published over the last decade, offering a thorough evaluation of the efficacy, effectiveness, and safety of silodosin. By incorporating observational studies, it provides a well-rounded perspective on the drug’s real-world effectiveness and tolerability. The limitations of this review include the use of only one database to perform the literature search, resulting in the inclusion of 23 studies. In addition, the small sample sizes and limited follow-up periods in many studies may reduce the generalizability of the findings. Inconsistencies in study protocols, particularly in the patient inclusion/exclusion criteria and the definition of clinical outcomes, could impact the validity of cross-study comparisons.

## Conclusions

The recent clinical evidence summarized in this review confirms that silodosin is a highly efficacious/effective and well-tolerated option for managing BPH and alleviating LUTS. According to EAU, AUA, and USI guidelines, clinicians should offer α1-blockers such as silodosin as a treatment option for patients with bothersome, moderate-to-severe LUTS/BPH. In addition, silodosin demonstrated an enhanced cardiovascular safety profile compared with other α1-blockers. Its selective targeting of α-1A adrenoceptors minimizes the risk of cardiovascular adverse events, making it a potentially suitable option for BPH patients, particularly in elderly patients with comorbidities.
